# Cigarette smoking increases the risk of nasopharyngeal carcinoma through the elevated level of IgA antibody against Epstein‐Barr virus capsid antigen: A mediation analysis

**DOI:** 10.1002/cam4.2832

**Published:** 2020-01-10

**Authors:** Wan‐Lun Hsu, Yin‐Chu Chien, Yen‐Tsung Huang, Kelly J. Yu, Jenq‐Yuh Ko, Ching‐Yuan Lin, Yung‐An Tsou, Yi‐Shing Leu, Li‐Jen Liao, Yen‐Liang Chang, Jia‐Ying Su, Zhiwei Liu, Cheng‐Ping Wang, Shyuang‐Der Terng, Chun‐Hung Hua, Jehn‐Chuan Lee, Tsung‐Lin Yang, Chu‐Hsing Kate Hsiao, Ming‐Shiang Wu, Ming‐Hsui Tsai, Ming‐Jiung Liu, Pei‐Jen Lou, Allan Hildesheim, Chien‐Jen Chen, the GEV‐NPC Study Group

**Affiliations:** ^1^ Genomics Research Center Academia Sinica Taipei Taiwan; ^2^ Institute of Statistical Science Academia Sinica Taipei Taiwan; ^3^ Division of Cancer Epidemiology and Genetics National Cancer Institute Bethesda Maryland USA; ^4^ Department of Otolaryngology National Taiwan University Hospital and College of Medicine National Taiwan University Taipei Taiwan; ^5^ Department of Head and Neck Surgery Koo Foundation Sun Yat‐Sen Cancer Center Taipei Taiwan; ^6^ Department of Otorhinolaryngology China Medical University Hospital Taichung Taiwan; ^7^ Department of Otolaryngology MacKay Memorial Hospital Taipei Taiwan; ^8^ Department of Otolaryngology Far Eastern Memorial Hospital New Taipei City Taiwan; ^9^ Department of Otolaryngology Cathay General Hospital Taipei Taiwan; ^10^ Graduate Institute of Epidemiology and Preventative Medicine College of Public Health National Taiwan University Taipei Taiwan; ^11^ Department of Internal Medicine National Taiwan University Hospital Taipei Taiwan; ^12^ Department of Radiation Oncology Koo Foundation Sun Yat‐Sen Cancer Center Taipei Taiwan

**Keywords:** case‐control study, cigarette smoking, Epstein‐Barr Virus, mediation analysis, nasopharyngeal carcinoma

## Abstract

**Background:**

The study aims are to evaluate the associations between nasopharyngeal carcinoma (NPC) risk and cigarette smoking and to explore the effects of cigarette smoking on Epstein‐Barr virus (EBV) infection for NPC risk.

**Methods:**

1235 male NPC cases and 1262 hospital‐based male controls matched to cases were recruited across six collaborative hospitals between 2010 and 2014. Using a standardized questionnaire, information on cigarette smoking and other potential risk factors for NPC was obtained. Blood was collected and used for anti‐EBV VCA IgA and anti‐EBV EA‐EBNA1 IgA testing using standard methods. Unconditional logistic regression analysis was used to estimate odds ratio (OR) with 95% confidence interval (CI) for each risk factor after adjusting for confounders.

**Results:**

63.6% of cases and 44.0% of controls reported ever smoking cigarettes. After full adjustment, current smokers had a significant 1.60‐fold (95% CI = 1.30‐1.97) and former smokers a borderline significant 1.27‐fold (95% CI = 1.00‐1.60) increased NPC risk compared to never smokers. NPC risk increased with increasing duration, intensity, and pack‐years of cigarette smoking but not with age at smoking initiation. Among controls, anti‐EBV VCA IgA seropositivity rate was higher in current smokers than never smokers (14.0% vs 8.4%; OR = 1.82; 95% CI = 1.19‐2.79). Mediation analyses showed that more than 90% of the cigarette smoking effect on NPC risk is mediated through anti‐EBV VCA IgA.

**Conclusion:**

This study confirms the association between long‐term cigarette smoking and NPC and demonstrates that current smoking is associated with seropositivity of anti‐EBV VCA IgA antibodies.

## Graphical abstract

Smoking duration and intensity but not age at initiation are NPC risk factors, and anti‐EBV VCA IgA seropositivity rate was higher in current than never smokers among controls. Mediation analyses show that NPC and EBV association may be explained by the interplay between smoking and EBV antibodies serostatus.

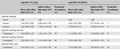

## 1 INTRODUCTION

Nasopharyngeal carcinoma (NPC) is a cancer linked to infection with the Epstein‐Barr virus (EBV),^1^, ^2^ which account for over 90% of NPC cases in regions of the world with high incidence of disease.^3^

EBV is a ubiquitous infection that typically occurs during childhood and adolescence leading to lifelong infection in over 90% of adults worldwide.^4^ In Taiwan, infection with EBV and seroconversion occurs before 10 years of age for 80% of the population and by 20 years of age for 96.2% of the population.^5^ Consistent with early exposure to its primary causal factor, NPC typically occurs at a younger age than most other adult solid tumors, with a mode age at diagnosis in the mid‐40s to early 50s. In regions of the world where NPC is common, incidence rates peak in the mid‐40s and plateau thereafter, with rates declining at older ages in some populations.^6^, ^7^ In addition to its necessary cause (EBV), a NPC family history and some inherited genetic polymorphisms (particularly in genes involved in immune response of infections including HLA genes), consumption of nitrosamines high food and its precursors (including salted fish), cigarette smoking, and possibly occupational exposure to wood dusts and formaldehyde are associated with NPC risk.^8^, ^9^ Interestingly, many of these factors/exposures are present at an early age as is EBV.^5^, ^10^ Inherited genetic factors are present at birth. Salted fish consumption and other salted/preserved foods associated with NPC are associated with stronger disease risk with early childhood exposure.^11^, ^12^, ^13^, ^14^ This pattern suggests that early life exposures are important for NPC development.

Cigarette smoking has been shown to be associated with NPC risk in several studies.^15^, ^16^, ^17^, ^18^ Unlike other cancers associated with smoking, most studies have found robust and significant associations in long‐term smokers of more than 15‐20 years only.^15^, ^16^ Many long‐term smokers who develop NPC start smoking during adolescence, making it difficult to determine whether long‐term use and/or early exposure explain observed associations. Given the known immunosuppressive effect of smoking,^19^, ^20^ one might speculate that smoking at early ages, in the initial years following primary EBV infection might predispose to NPC. Indirect support for this hypothesis includes the observation in a few studies that cigarette smoking is associated with increased levels of anti‐EBV IgA antibodies in those associated with increased NPC risk (VCA and EBNA1, for example)^17^ and that elevated anti‐EBV IgA antibodies are indicative of mucosal exposure to EBV during lytic viral reactivation of the virus in the pharyngeal space.^21^, ^22^, ^23^, ^24^

To further evaluate this question, we conducted a multicenter case‐control study of NPC in Taiwan to evaluate (a) patterns of smoking associated with risk, (b) effects of smoking on EBV antibody responses, and (c) immune modulatory effects of EBV on smoking and NPC risk.

## 2 MATERIALS AND METHODS

### 2.1 Study population

A multicenter hospital‐based case‐control study was conducted in Northern and Central Taiwan. Cases and controls were recruited from six collaborative hospitals including Cathay General Hospital, China Medical University Hospital, Far‐Eastern Memorial Hospital, Koo Foundation Sun Yat‐Sen Cancer Center, Mackay Memorial Hospital, and National Taiwan University Hospital. Subjects were recruited between July 2010 and December 2014. Institutional Review Boards at the Academia Sinica, National Cancer Institute in the United States, and collaborative hospitals approved the study protocol and informed consent. Eligibility criteria for NPC cases and controls included age greater than 18 years, no previous diagnosis and/or treatment for cancers, and residence in Northern/Central Taiwan at least 6 months prior to NPC diagnosis or recruitment. NPC cases (International Classification of Diseases, Ninth Revision code 147) were histologically confirmed and first diagnosed between 2007 and 2014. Cases diagnosed between July 2010 and December 2014 were recruited at the time of diagnosis and prior to treatment initiation (prospective ascertainment). Cases diagnosed between January 2007 and June 2010 were also recruited post‐treatment (retrospective ascertainment). The cutoff of January 2007 was chosen for retrospective ascertainment to minimize survival bias; 3‐year NPC survival in Taiwan is approximately 75%.^25^ Controls from Northern and Central Taiwan (the ascertainment area covered by participating hospitals that recruited NPC cases) were recruited via the Health Examination Centers or ENT wards/outpatient clinics in the participating centers and matched to cases on age (±5 years) and sex. 1873 eligible NPC cases and 1885 controls were identified, of which 1623 NPC cases (86.7%) and 1804 controls (95.7%) agreed to participate and were recruited. Among NPC cases, 892 were prospectively ascertained and 731 retrospectively ascertained.

### 2.2 Data collection

Written consent was obtained from study participants. Participants were interviewed face to face by trained nurses using a structured questionnaire that obtained information on sociodemographic characteristics and other exposures of interest. Cigarette smoking habit was defined as having smoked regularly for at least 6 months. Information was collected on ever smoking status (at the time of diagnosis for NPC cases and recruitment for controls). For cigarette smokers, age at initiation, age at cessation, duration and quantity of cigarettes were also recorded. Former smokers were defined as subjects who quit smoking more than 1 year before NPC diagnosis or interview date for cases and controls, respectively.

### 2.3 Specimen collection and testing

Biological samples including 20‐30 mL blood were obtained from 1525 cases (94.0% of participants) and 1771 controls (98.2% of participants), processed by centrifugation and frozen at −80°C within 24 hours of collection for 80% of participants and within 48 hours for 90%. Resultant plasma was used for EBV antibody testing of 817 prospective NPC cases and 1765 controls. We tested for anti‐EBV VCA IgA (EUROIMMUN, Germany) and anti‐EBV EA‐EBNA1 IgA (MeDiPro, Formosa Biomedical Technology Corp, Taiwan) by ELISA per manufacturer protocol. The manufacturer defined cutoff of 1.0 EU/mL was used to define positivity for VCA IgA. The EA‐EBNA1 IgA kit measured IgA against the early antigen combined with Epstein‐Barr nuclear antigen‐1 previously shown to have a sensitivity of 94.2% and specificity of 82.6% with an area under the receiver operating characteristic of 96.4 for the detection of NPC.^26^ We defined the EA‐EBNA1 IgA cutoff for purposes of our analysis (3.0 EU/mL) as the value that maximized the sensitivity + specificity to distinguish NPC cases from controls (ie, greatest Youden index) since no manufacturer suggested positivity cutoff was provided (only a cutoff defining clinically aberrant antibody levels was provided for this assay). Approximately 10% of specimens were randomly selected for blinded replicate testing to assess assay reproducibility. The resultant percent agreement for classifying individuals as positive versus negative was 94% for anti‐EBV VCA IgA and 96% for anti‐EBV EA‐EBNA1 IgA. Agreement when restricting to those with at least one seropositive result observed (agreement among positives) was 77% for anti‐EBV VCA IgA and 63% for anti‐EBV EA‐EBNA1 IgA.

### 2.4 Statistical analysis

We restricted our analysis to males given the small number of females reporting a history smoking in the study (8.7% of 930 females vs 53.7% of 2497 males). The association between smoking and NPC among females is summarized in Table S1 for completeness. We noted that retrospectively and prospectively ascertained cases were similar with respect to age, gender, education, family history, and smoking distribution (Table S2); we therefore combined them in the analysis. To evaluate the association between smoking (never, former, current) and patterns of smoking (duration, intensity, pack‐years, and age at start) and NPC as well as to evaluate the association between smoking and anti‐EBV antibody seropositivity, unconditional logistic regression models were used to estimate odds ratios (ORs) and corresponding 95% confidence intervals (CIs). To evaluate the association between smoking and patterns of smoking and NPC, we present models adjusted for age and study region only and models that additionally adjust for ethnicity, education and family history of NPC. Heterogeneity of effects by duration and intensity was evaluated by including an interaction term in the models. To evaluate the association between smoking and anti‐EBV antibody seropositivity, we present models adjusted for age only, since age was the only parameter associated with EBV seropositivity. Trend tests were performed by treating categorical variables as continuous both overall and restricted to ever smokers for both analyses. Direct and indirect effects of the cigarette smoking on NPC risk in relation to EBV infection were evaluated using mediation analyses.^27^, ^28^ Two regression models were required to perform mediation analyses: a model for the mediator (anti‐EBV antibody seropositivity) determined by the exposure (cigarette smoking), and a model for the NPC risk determined jointly by the exposure and the mediator. CIs of direct and indirect effects were estimated from 5000 bootstrapping replicates. The detailed methodology of mediation analyses can be found elsewhere.^29^

## 3 RESULTS

This analysis included 1235 male NPC cases and 1262 male controls. The distribution of cases and controls with respect to age and other sociodemographic factors is presented in Table 1. The median age of cases and controls was 48.4 and 48.6, respectively. 83.8% of both study groups resided in Northern Taiwan. Cases were less educated than controls (median years of education: 12 for cases; 16 for controls) and slightly more likely than controls to report being of Hakka origin (13.8% of cases; 9.4% of controls). Cases were more likely than controls to report a family history of NPC (13.9% of cases; 4.3% of controls) but were equally likely to report having consumed salted fish at a young age (4.2% of cases; 5.3% of controls).

**Table 1:** Characteristics of male NPC cases and controls—NPC multicenter case‐control study in Taiwan

Risk factor	# Cases (%)	# Controls (%)
Age		
Median	48.4	48.6
<40	286 (23.1)	308 (24.4)
40‐49	421 (34.1)	361 (28.6)
50‐59	359 (29.1)	356 (28.2)
60‐69	169 (13.7)	237 (18.8)
Study region^a^		
Northern Taiwan	970 (78.7)	1122 (89.4)
Central Taiwan	262 (21.3)	133 (10.6)
Education years		
Median	12.0	16.0
<=9	348 (28.2)	141 (11.2)
10‐12	563 (45.6)	342 (27.1)
>12	324 (26.2)	779 (61.7)
Ethnicity^b^		
Taiwanese	887 (72.0)	906 (71.9)
Hakka	170 (13.8)	119 ( 9.4)
Mainlander	164 (13.3)	228 (18.1)
Others	11 ( 0.9)	8 ( 0.6)
Family History of NPC		
No	1063 (86.1)	1208 (95.7)
Yes	172 (13.9)	54 (4.3)
Salted Fish @ Young Age^c^		
No	1140 (95.8)	1127 (94.7)
Yes	50 (4.2)	63 (5.3)
Total	1235	1262

a Data for study region were not available for 3 cases and 7 controls.

b Data for ethnicity were missing for 3 cases and 1 control.

c Data for salted fish at young age were missing for 45 cases and 72 controls.

### 3.1 Association between cigarette smoking and NPC

Results evaluating the association between patterns of cigarette smoking and NPC are summarized in Table 2. 41.8% of cases and 24.1% of controls reported being current smokers, while 21.8% of cases and 19.9% of controls reported former smoking. After full adjustment, current smokers had a significant 1.60‐fold (95% CI = 1.30‐1.97) increased NPC risk compared to nonsmokers. Risk was marginally increased among former smokers (OR = 1.27; 95% CI = 1.00‐1.60), and no specific risk pattern was observed evaluating time since smoking cessation.

**Table 2:** Association between smoking and NPC among males—NPC multicenter case‐control study in Taiwan

Risk factor	# Cases (%)	# Controls (%)	Partially Adjusted^a^		Fully Adjusted^b^	
			OR	95% CI	OR	95% CI
Cigarette smoking						
Never	450 (36.4)	707 (56.0)	1.00		1.00	
Former	269 (21.8)	251 (19.9)	1.62	1.31‐2.01	1.27	1.00‐1.60
Quit > 10 y ago	137 (11.1)	134 (10.6)	1.53	1.16‐2.02	1.25	0.93‐1.69
Quit 6‐10 y ago	51 ( 4.1)	61 ( 4.8)	1.30	0.88‐1.93	1.02	0.67‐1.56
Quit <=5 y ago	81 ( 6.6)	56 ( 4.5)	2.17	1.51‐3.13	1.54	1.04‐2.29
Current	516 (41.8)	304 (24.1)	2.60	2.16‐3.14	1.60	1.30‐1.97
Cigarette smoking duration^c^						
Never	450 (36.5)	707 (56.0)	1.00		1.00	
<=10 y	72 ( 5.8)	105 ( 8.3)	0.99	0.71‐1.37	0.76	0.53‐1.08
11‐20 y	203 (16.4)	142 (11.3)	2.06	1.61‐2.65	1.44	1.10‐1.89
>20 y	509 (41.3)	308 (24.4)	2.65	2.19‐3.21	1.72	1.39‐2.13
p‐trend overall/ among ever smokers			<0.01/ <0.01		<0.01/ <0.01	
Cigarette smoking intensity^d^						
Never	450 (36.5)	707 (56.1)	1.00		1.00	
<= 15	255 (20.7)	282 (22.4)	1.40	1.14‐1.73	1.09	0.87‐1.36
16‐25	339 (27.5)	188 (14.9)	2.78	2.24‐3.46	1.77	1.39‐2.24
>25	189 (15.3)	83 ( 6.6)	3.43	2.58‐4.57	1.98	1.45‐2.70
p‐trend overall/ among ever smokers			<0.01/ <0.01		<0.01/ <0.01	
Cigarette Pack‐yrs^e^						
Never	450 (36.5)	707 (56.1)	1.00		1.00	
<=10	155 (12.6)	180 (14.3)	1.28	0.99‐1.64	1.01	0.77‐1.33
11‐20	188 (15.3)	155 (12.3)	1.85	1.45‐2.37	1.25	0.96‐1.64
>20	439 (35.6)	218 (17.3)	3.21	2.61‐3.94	1.98	1.58‐2.49
p‐trend overall/ among ever smokers			<0.01/ <0.01		<0.01/ <0.01	
Cigarette age at start^f^						
Never	450 (36.5)	707 (56.1)	1.00		1.00	
30+	24 ( 1.9)	15 ( 1.2)	2.65	1.36‐5.15	1.68	0.82‐3.43
20‐29	200 (16.2)	145 (11.5)	2.15	1.68‐2.76	1.72	1.31‐2.25
<20	560 (45.4)	394 (31.2)	2.16	1.81‐2.58	1.36	1.11‐1.66
p‐trend overall/ among ever smokers			<0.01/ 0.87		<0.01/ 0.17	

a Adjusted for age and study region.

b Adjusted for age, study region, ethnicity, education, and family history of NPC.

c Data for cigarette smoking duration were missing for 1 case.

d Data for cigarette smoking intensity were missing for 2 cases and 2 controls.

e Data for cigarette pack‐yrs were missing for 3 cases and 2 controls.

f Data for cigarette age at start were missing for 1 case and 1 control.

NPC risk increased within increasing duration (p‐trend < 0.01 overall and < 0.01 among ever smokers), intensity (p‐trend < 0.01 overall and < 0.01 among ever smokers), and pack‐years (p‐trend < 0.01 overall and < 0.01 among ever smokers) of smoking. While a significant trend was observed when age at smoking initiation was evaluated overall (p‐trend < 0.01 overall), no trend remained among ever smokers (*P* = .17 among ever smokers) indicating that the significant trend observed overall was due to differences between never and ever smokers and that there was no evidence for a trend with decreasing age at smoking initiation among smokers. Compared to never smokers, significantly elevated NPC risk was observed for those who smoked for 11‐20 years (OR = 1.44; 95% CI = 1.10‐1.89) and > 20 years (OR = 1.72; 95% CI = 1.39‐2.13), for those who smoked 16‐25 cigarettes per day (OR = 1.77; 95% CI = 1.39‐2.24) and > 25 cigarettes per day (OR = 1.98; 95% CI = 1.45‐2.70), and for those who smoked > 20 pack‐years (OR = 1.98; 95% CI = 1.58‐2.49). When smoking duration and intensity were evaluated jointly (Table 3), risk was found to increase with increasing duration within strata of intensity and with increasing intensity within strata of duration with no evidence for statistical heterogeneity (p‐het = 0.13).

**Table 3:** Joint association between smoking duration/intensity and NPC among males—NPC multicenter case‐control study in Taiwan

Risk Factor		# Cases (%)	# Controls (%)	Partially Adjusted^a^		Fully Adjusted^b^	
				OR	95% CI	OR	95% CI
Cigarette Smoking Duration	Cigarette Smoking Intensity						
Never	Never	450 (36.5)	707 (56.1)	1.00		1.00	
<=10 y	<=15 cigarettes	47 ( 3.8)	75 ( 5.9)	0.91	0.62‐1.35	0.74	0.49‐1.12
<=10 y	16‐25 cigarettes	16 ( 1.3)	24 ( 1.9)	0.97	0.51‐1.87	0.66	0.33‐1.36
<=10 y	>25 cigarettes	8 ( 0.7)	5 ( 0.4)	2.23	0.70‐7.07	1.83	0.51‐6.52
11‐20 y	<=15 cigarettes	76 ( 6.2)	76 ( 6.0)	1.48	1.05‐2.09	1.13	0.78‐1.63
11‐20 y	16‐25 cigarettes	90 ( 7.3)	50 ( 4.0)	2.61	1.80‐3.78	1.68	1.12‐2.50
11‐20 y	>25 cigarettes	37 ( 3.0)	16 ( 1.3)	3.07	1.67‐5.65	2.11	1.11‐4.03
>20 y	<=15 cigarettes	132 (10.7)	131 (10.4)	1.65	1.26‐2.17	1.28	0.95‐1.72
>20 y	16‐25 cigarettes	232 (18.8)	114 ( 9.1)	3.27	2.52‐4.24	2.05	1.55‐2.72
>20 y	>25 cigarettes	144 (11.7)	62 ( 4.9)	3.66	2.64‐5.06	1.98	1.40‐2.81

Note

Data were missing for 3 cases and 2 controls.

a Adjusted for age and study region.

b Adjusted for age, study region, ethnicity, education, and family history of NPC.

### 3.2 Cigarette smoking and EBV seroreactivity

9.8% and 10.9% of male controls in our study were seropositive for anti‐EBV VCA IgA and anti‐EBV EA‐EBNA1 IgA, respectively (Table 4). Among seropositives, anti‐EBV VCA IgA and anti‐EBV EA‐EBNA1 IgA median levels were 1.46 (range: 1.00‐7.69) and 4.08 (range: 3.01‐15.11). Anti‐EBV VCA IgA and anti‐EBV EA‐EBNA1 IgA seropositivity was not associated with sociodemographic (age, study region, education, ethnicity) or other (family history of NPC, salted fish consumption) variables evaluated, with one exception (Table S3); men under 40 years old were more likely to be seropositive (18.7%) than men in their 40s, 50s, or 60s (7.1%‐9.2%, *P* < .0001). For subsequent analyses reported below, we therefore adjusted for age.

**Table 4:** Association between EBV and smoking among male controls—NPC multicenter case‐control study in Taiwan

Risk factor	Anti‐EBV VCA IgA				Anti‐EBV EA‐EBNA1 IgA			
	# Positive (%)	# Negative (%)	OR	95% CI	# Positive (%)	# Negative (%)	OR	95% CI
Cigarette smoking								
Never	58 ( 8.4)	634 (91.6)	1.00		80 (11.5)	614 (88.5)	1.00	
Former	21 (8.4)	228 (91.6)	0.97	0.57‐1.64	18 ( 7.2)	231 (92.8)	0.70	0.41‐1.21
Quit > 10 y ago	10 ( 7.5)	124 (92.5)	1.00		12 ( 9.0)	122 (91.0)	1.00	
Quit 6‐10 y ago	5 ( 8.3)	55 (91.7)	1.29	0.40‐4.16	5 ( 8.3)	55 (91.7)	0.49	0.14‐1.66
Quit <=5 y ago	6 (10.9)	49 (89.1)	1.75	0.57‐5.39	1 (1.8)	54 (98.2)	0.08	0.01‐0.71
p‐trend			0.33				0.01	
Current	42 (14.0)	258 (86.0)	1.82	1.19‐2.79	37 (12.3)	264 (87.7)	1.01	0.66‐1.53
<=10 y	4 (12.9)	27 (87.1)	1.00		4 (12.9)	27 (87.1)	1.00	
11‐20 y	10 (12.7)	69 (87.3)	0.84	0.24‐2.95	15 (19.0)	64 (81.0)	1.52	0.45‐5.12
>20 y	28 (14.7)	162 (85.3)	0.58	0.15‐2.34	18 ( 9.4)	173 (90.6)	0.60	0.14‐2.68
p‐trend			0.40				0.36	
<=15 cigarettes	17 (10.9)	139 (89.1)	1.00		18 (11.5)	138 (88.5)	1.00	
16‐25 cigarettes	15 (15.3)	83 (84.7)	1.39	0.65‐2.95	8 ( 8.1)	91 (91.9)	0.73	0.30‐1.77
>25 cigarettes	10 (22.2)	35 (77.8)	2.14	0.89‐5.14	11 (24.4)	34 (75.6)	2.83	1.19‐6.75
p‐trend			0.09				0.06	
Total	121 ( 9.8)	1120 (90.2)			135 (10.9)	1109 (89.1)		

Note

Adjusted for age.

Data for cigarette smoking intensity were missing for 1 subjects.

When anti‐EBV VCA IgA was examined, we noted similar seropositivity rates for never and former smokers (8.4% and 8.4%, respectively; OR = 0.97; 95% CI = 0.57‐1.64) (Table 4). Among former smokers, no significant trend was observed by time since quitting, although seropositivity was slightly higher among those who quit smoking within 5 years (10.9%; OR = 1.75 compared to those who quit >10 years ago; 95% CI = 0.57‐5.39). In contrast, anti‐EBV VCA IgA seropositivity was significantly higher among current smokers compared to never smokers (14.0%; OR = 1.82; 95% CI = 1.19‐2.79), and among current smokers, seropositivity increased with increasing intensity of smoking (p‐trend = 0.09) but not with increasing duration of smoking (p‐trend = 0.40). As summarized in Table S4, among anti‐EBV VCA IgA‐seropositive individuals, antibody levels were comparable among never, former, and current smokers. No evidence for a significant association between smoking and anti‐EBV EA‐EBNA1 IgA was noted (Table 4 and Table S4).

### 3.3 Mediation analyses of cigarette smoking, EBV seroreactivity, and NPC risk

There were 569 (88.1%) and 584 (90.4%) prospective male NPC cases seropositive for anti‐EBV VCA IgA and anti‐EBV EA‐EBNA1 IgA, respectively. Compared with seropositive controls, OR_adj_ (95% CI) of NPC was 65.56 (47.40‐90.68) and 91.19 (63.48‐130.99), respectively, for anti‐EBV VCA IgA and anti‐EBV EA‐EBNA1 IgA. Mediation analyses were conducted to further clarify the roles of cigarette smoking and EBV in the development of NPC. Table 5 shows that compared to never smokers, current smokers had an increased NPC risk mediated through anti‐EBV VCA IgA (indirect risk ratio (RR) = 1.56, 95% CI = 1.12‐2.23 and direct RR = 1.02, 95% CI = 0.70‐1.50). Under the risk ratio scale, more than 90% of current smoking's effect on NPC risk was mediated through anti‐EBV VCA IgA. A significant dose response was also observed for the indirect effects by intensity, duration, and cumulative exposure of cigarette smoking. In contrast, smoking did not appear to have a mediation effect on NPC risk via anti‐EBV EA‐EBNA1 IgA.

**Table 5:** Mediation analyses for cigarette smoking and anti‐EBV antibodies and NPC risk

	Anti‐EBV VCA IgA				Anti‐EBV EA‐EBNA1 IgA		
	Direct effect Risk Ratio (95% CI)	Indirect effect Risk Ratio (95% CI)	Proportion of mediation (%)	Direct effect Risk Ratio (95% CI)		Indirect effect Risk Ratio (95% CI)	Proportion of mediation (%)
Cigarette smoking							
Never	1.00	1.00		1.00		1.00	
Former	1.36 (0.91‐2.09)	0.99 (0.64‐1.46)	—	1.66 (1.09‐2.60)		0.77 (0.46‐1.14)	—
Current	1.02 (0.69‐1.50)	1.56 (1.12‐2.24)	96	1.39 (0.94‐2.11)		1.03 (0.72‐1.47)	9
Cigarette smoking intensity							
Continuous^a^	1.01 (0.88‐1.15)	1.22 (1.07‐1.38)	95	1.11 (0.98‐1.29)		1.05 (0.91‐1.20)	33
Cigarette smoking duration							
Continuous^b^	1.01 (0.91‐1.12)	1.14 (1.04‐1.26)	94	1.10 (1.00‐1.23)		1.01 (0.91‐1.12)	12
Cigarette Pack‐years							
Continuous^c^	1.01 (0.91‐1.12)	1.17 (1.05‐1.29)	93	1.11 (1.00‐1.26)		1.02 (0.91‐1.14)	18

Note

Adjusted for age, study region, ethnicity, education, and family history of NPC.

a The continuous variable is categorized as never, former, <=15 cigarettes, 16‐25 cigarettes, >25 cigarettes.

b The continuous variable is categorized as never, former, <=10 y, 11‐20 y, >20 y.

c The continuous variable is categorized as never, former, <=10 pack‐years, 11‐20 pack‐years, >20 pack‐years.

## 4 DISCUSSION

Studies have consistently found that cigarette smoking is associated with NPC risk^3^, ^30^ but it remains unclear what smoking parameters (duration, intensity or age at initiation) are important to confer risk. Whether smoking increases NPC risk partly through immune modulatory effects on EBV is also not fully understood.^17^ In this study, we confirm that smoking increases NPC risk and suggest that duration and intensity of smoking, rather than age at smoking initiation, are the primary risk factors of smoking. We also show that current cigarette smoking is associated with increases in anti‐EBV VCA IgA seropositivity, and that among current smokers, seropositivity increases with increased intensity rather than smoking duration, indicative of an acute rather than long‐term effect on antibody responses to EBV. In addition, mediation analysis in our study reveals that the effect of smoking on NPC risk is mostly mediated through EBV infection measured by anti‐EBV VCA IgA. To our knowledge, this is the first study to evaluate the role of cigarette smoking on anti‐EBV antibody seropositivity associated with NPC risk.

The a priori hypothesis of this analysis is that exposures at early ages are of relevance to NPC pathogenesis and that early age at smoking initiation would be an important predictor of NPC risk. Our findings, however, do not support this a priori hypothesis given that duration and intensity of cigarette smoking, rather than age at smoking initiation, were more consistently associated with NPC risk. It warrants mention, however, that the vast majority of smokers who developed NPC (560 out of 784 or 71.4%) started smoking prior to the age of 20 and that given the young median age of NPC diagnosis (48.4 years in our study), many long‐term smokers also initiated smoking early in life making it difficult to separate the effect of early initiation and duration of smoking. Future meta or pooled analyses across multiple large, well‐controlled studies will need to evaluate this question with greater statistical power.

A second objective of our study was to explore whether cigarette smoking affects host responses to EBV infection. Since nearly everyone is infected with EBV with lifelong infection in its host, presence of elevations in antibodies targeting EBV is likely a measure of level of exposure.^31^, ^32^ We further focused on IgA measurements since these antibodies are typically generated in response to mucosal exposures and likely to reflect exposure at the nasopharynx.^16^, ^21^, ^22^, ^23^, ^24^ Our finding that current smoking (but not former smoking) is associated with higher seropositivity rates against anti‐EBV VCA IgA and that seropositivity rates increased with increasing intensity of use among current smokers suggest that smoking has an acute effect on the host ability to control EBV infections, leading to higher virus exposure at mucosal surfaces and therefore higher IgA seropositivity rates. This finding is consistent with previous reports from China and Taiwan in which current smokers had a higher anti‐EBV VCA IgA seropositivity risk than former smokers and never smokers.^17^, ^33^ No association between smoking and anti‐EBV EA‐EBNA1 IgA was noted in the present study. In contrast to our findings, a study conducted in an NPC endemic area in China reported a significant association between anti‐EBV EBNA1 IgA and smoking^34^ These differing findings might be explained by the fact that the study from China and ours tested for antibodies targeting different EBV antigens, with our study using a test that detects antibodies against both early lytic (EA) and latent (EBNA‐1) phase proteins while the China study detected antibodies against a latent phase protein only (EBNA‐1).

The third objective of the study was to use mediation analysis to better understand the role of smoking and EBV in NPC carcinogenesis. If an association exists between smoking and the antibodies measured levels targeting EBV antigens, one possible biological explanation for the association between smoking and NPC might be its effect on the host ability to control EBV infection. Our finding showed that smoking affects NPC risk mediated by anti‐EBV VCA IgA but not by anti‐EBV EA‐EBNA IgA. One possible explanation for this apparent discrepant finding is that, unlike VCA, which is produced in the late lytic phase of EBV infection (ie, in its final stages when the virus has replicated in the host and viral particles are being formed), EBNA1, the primary antigen targeted by the anti‐EBV EA‐EBNA1 IgA assay used, is produced during the latency phase of EBV infection and is therefore less likely to reflect mucosal exposure.^35^, ^36^

A strength of our study is its multicenter nature to recruit a considerable and likely representative set of NPC cases from Taiwan. The study is not without limitations, including the small number of female smokers that precluded evaluation of these analyses among females and the inclusion of retrospective ascertainment of NPC cases which could potentially lead to survival biases. To mitigate this later limitation, we restricted retrospective NPC cases to those diagnosed within 3 years of study start since 3‐year survival following NPC diagnosis is high in Taiwan and survival bias therefore likely not of major concern.^25^ Another consideration is temporality. Given the case‐control design, samples for cases were collected at the time of diagnoses. To better evaluate the role of anti‐EBV antibody seromarkers and its mediation effect on NPC risk, a cohort design would be preferable.

In summary, we conducted a study to evaluate patterns of association between cigarette smoking and NPC and to examine whether smoking has an effect on EBV antibodies. We noted that duration and intensity of smoking but not age at smoking initiation are risk factors for NPC and demonstrate that current smoking is associated with elevations in anti‐EBV VCA IgA antibodies thus providing a possible mechanism by which smoking increases NPC risk by modulating host responses to EBV infection.

## CONFLICT OF INTEREST

The authors declare no potential conflicts of interest.

## AUTHOR CONTRIBUTIONS

Wan‐Lun Hsu: Writing–original draft, project administration, investigation, quality control of data and algorithms, data analysis and interpretation, statistical analysis, and final approval of the version to be published. Yin‐Chu Chien: Project administration, investigation, quality control of data and algorithms, data analysis and interpretation, and final approval of the version to be published. Yen‐Tsung Huang: Investigation, data analysis and interpretation, statistical analysis, and final approval of the version to be published. Kelly J. Yu: Project administration, investigation, quality control of data and algorithms, data analysis and interpretation, and final approval of the version to be published. Jenq‐Yuh Ko, Ching‐Yuan Lin, Yung‐An Tsou, Yi‐Shing Leu, Li‐Jen Liao, Yen‐Liang Chang, Cheng‐Ping Wang, Shyuang‐Der Terng, Chun‐Hung Hua, Jehn‐Chuan Lee, Tsung‐Lin Yang, Ming‐Shiang Wu, Ming‐Hsui Tsai, Ming‐Jiung Liu: Data acquisition, investigation, and final approval of the version to be published. Jia‐Ying Su: Investigation, statistical analysis, and final approval of the version to be published. Zhiwei Liu: Investigation, quality control of data and algorithms, data analysis and interpretation, and final approval of the version to be published. Chu‐Hsing Hsiao: Investigation, quality control of data and algorithms, and final approval of the version to be published. Pei‐Jen Lou: Data acquisition, investigation and final approval of the version to be published. Allan Hildesheim: Investigation, data analysis and interpretation, and final approval of the version to be published. Chien‐Jen Chen: Conception and design, funding acquisition, investigation, quality control of data and algorithms, data analysis and interpretation, and final approval of the version to be published.
