# Supplementary material for: Cigarette smoking increases the risk of nasopharyngeal carcinoma through the elevated level of IgA antibody against Epstein‐Barr virus capsid antigen: A mediation analysis
Source: Cancer Med. 2020 Jan 10;9(5):1867–76. doi: 10.1002/cam4.2832 (PMC7050088; doi:10.1002/cam4.2832)
Supplement: Supplementary file 1 [file CAM4-9-1867-s001.docx]

Supplemental Table 1. Association Between Smoking and NPC Among Female - NPC Multicenter Case Control Study in Taiwan

| Risk Factor | # Cases (%) | # Controls (%) | Partially Adjusted ^†^ | | Fully Adjusted ^‡^ | |
| --- | --- | --- | --- | --- | --- | --- |
|  |  |  | OR | 95% CI | OR | 95% CI |
| Cigarette Smoking |  |  |  |  |  |  |
| Never | 343 (88.4) | 506 (93.4) | 1.00 |  | 1.00 |  |
| Former | 9 ( 2.3) | 5 ( 0.9) | 2.57 | 0.85-7.78 | 1.93 | 0.59-6.33 |
| Current | 36 ( 9.3) | 31 ( 5.7) | 1.55 | 0.93-2.57 | 0.93 | 0.54-1.61 |
| Cigarette Smoking Duration ^§^ |  |  |  |  |  |  |
| Never | 343 (88.4) | 506 (93.5) | 1.00 |  | 1.00 |  |
| <=10 years | 8 ( 2.1) | 9 ( 1.7) | 1.16 | 0.44-3.08 | 0.77 | 0.27-2.14 |
| >10 years | 37 ( 9.5) | 26 ( 4.8) | 1.94 | 1.15-3.28 | 1.19 | 0.67-2.11 |
| **Total** | **388** | **542** |  |  |  |  |

^†^Adjusted for age and study region

^‡^Adjusted for age, study region, ethnicity, education and family history of NPC

^§^ Data for cigarette smoking duration was missing for 1 control

Supplemental Table 2. Association Between Smoking and NPC Stratified by Ascertainment Method (Retrospective vs. Prospective) (Males Only)

| Risk Factor | # Prospective NPC Cases (%) | # Retrospective NPC Cases (%) | # Controls (%) | Prospective | | | | Retrospective | | | |
| --- | --- | --- | --- | --- | --- | --- | --- | --- | --- | --- | --- |
|  |  |  |  | Adj OR ^†^ | 95% CI | Adj OR^‡^ | 95% CI | Adj OR ^†^ | 95% CI | Adj OR^‡^ | 95% CI |
| Cigarette Smoking |  |  |  |  |  |  |  |  |  |  |  |
| Never | 255 (36.4) | 195 (36.5) | 707 (56.0) | 1.00 |  | 1.00 |  | 1.00 |  | 1.00 |  |
| Former | 159 (22.7) | 110 (20.6) | 251 (19.9) | 1.64 | 1.27-2.10 | 1.29 | 0.99-1.69 | 1.58 | 1.19-2.10 | 1.22 | 0.90-1.66 |
| Quit >10 years ago | 84 (12.0) | 53 ( 9.9) | 134 (10.6) | 1.57 | 1.14-2.16 | 1.30 | 0.92-1.82 | 1.47 | 1.02-2.13 | 1.23 | 0.83-1.83 |
| Quit 6-10 years ago | 37 ( 5.3) | 14 ( 2.6) | 61 ( 4.8) | 1.64 | 1.06-2.54 | 1.27 | 0.80-2.01 | 0.84 | 0.46-1.54 | 0.67 | 0.35-1.27 |
| Quit <=5 years ago | 38 ( 5.4) | 43 ( 8.1) | 56 ( 4.5) | 1.77 | 1.14-2.75 | 1.31 | 0.82-2.10 | 2.58 | 1.67-4.00 | 1.71 | 1.06-2.75 |
| Current | 287 (40.9) | 229 (42.9) | 304 (24.1) | 2.53 | 2.04-3.15 | 1.62 | 1.27-2.06 | 2.68 | 2.12-3.40 | 1.59 | 1.22-2.08 |
| Cigarette Smoking Duration^§^ |  |  |  |  |  |  |  |  |  |  |  |
| Never | 255 (36.4) | 195 (36.5) | 707 (56.0) | 1.00 |  | 1.00 |  | 1.00 |  | 1.00 |  |
| <=5 years | 8 ( 1.1) | 20 ( 3.8) | 37 ( 2.9) | 0.55 | 0.25-1.20 | 0.46 | 0.20-1.05 | 1.77 | 0.99-3.18 | 1.41 | 0.76-2.63 |
| 6-10 years | 28 ( 4.0) | 16 ( 3.0) | 68 ( 5.4) | 1.04 | 0.65-1.66 | 0.82 | 0.50-1.34 | 0.72 | 0.40-1.29 | 0.49 | 0.26-0.92 |
| 11-15 years | 48 ( 6.9) | 34 ( 6.4) | 52 ( 4.1) | 2.36 | 1.54-3.62 | 1.63 | 1.04-2.56 | 2.05 | 1.27-3.30 | 1.28 | 0.76-2.16 |
| 16-20 years | 69 ( 9.9) | 52 ( 9.7) | 90 ( 7.1) | 1.96 | 1.38-2.79 | 1.45 | 1.00-2.12 | 1.93 | 1.31-2.84 | 1.32 | 0.87-2.02 |
| 21-25 years | 74 (10.6) | 55 (10.3) | 84 ( 6.7) | 2.39 | 1.69-3.38 | 1.77 | 1.22-2.57 | 2.46 | 1.68-3.60 | 1.74 | 1.14-2.64 |
| >25 years | 218 (31.1) | 162 (30.3) | 224 (17.8) | 2.68 | 2.09-3.42 | 1.72 | 1.31-2.25 | 2.91 | 2.22-3.83 | 1.74 | 1.29-2.35 |
| p-trend |  |  |  | <0.01 |  | <0.01 |  | <0.01 |  | <0.01 |  |
| Cigarette Smoking Intensity^¶^ |  |  |  |  |  |  |  |  |  |  |  |
| Never | 255 (36.5) | 195 (36.5) | 707 (56.1) | 1.00 |  | 1.00 |  | 1.00 |  | 1.00 |  |
| <= 5 | 32 ( 4.6) | 24 ( 4.5) | 52 ( 4.1) | 1.64 | 1.03-2.62 | 1.67 | 1.02-2.73 | 1.72 | 1.03-2.89 | 1.58 | 0.91-2.74 |
| 6-10 | 80 (11.4) | 60 (11.2) | 151 (12.0) | 1.42 | 1.05-1.94 | 1.08 | 0.77-1.50 | 1.43 | 1.01-2.01 | 1.04 | 0.72-1.51 |
| 11-15 | 37 ( 5.3) | 22 ( 4.1) | 79 ( 6.3) | 1.26 | 0.83-1.91 | 0.93 | 0.60-1.45 | 1.01 | 0.61-1.67 | 0.71 | 0.41-1.21 |
| 16-20 | 187 (26.8) | 139 (26.0) | 181 (14.4) | 2.76 | 2.14-3.55 | 1.76 | 1.33-2.32 | 2.77 | 2.10-3.66 | 1.74 | 1.28-2.36 |
| 21-25 | 8 ( 1.1) | 5 ( 0.9) | 7 ( 0.6) | 3.13 | 1.12-8.76 | 2.19 | 0.74-6.46 | 3.00 | 0.94-9.56 | 1.33 | 0.40-4.48 |

Supplemental Table 2. Association Between Smoking and NPC Stratified by Ascertainment Method (Retrospective vs. Prospective) (Males Only) (continued)

| >25 | 100 (14.3) | 89 (16.7) | 83 ( 6.6) | 3.15 | 2.27-4.36 | 1.87 | 1.31-2.67 | 3.79 | 2.69-5.35 | 2.04 | 1.39-2.98 |
| --- | --- | --- | --- | --- | --- | --- | --- | --- | --- | --- | --- |
| p-trend |  |  |  | <0.01 |  | <0.01 |  | <0.01 |  | <0.01 |  |
| Cigarette Pack-yrs^＆^ |  |  |  |  |  |  |  |  |  |  |  |
| Never | 255 (36.5) | 195 (36.5) | 707 (56.1) | 1.00 |  | 1.00 |  | 1.00 |  | 1.00 |  |
| <= 5 | 43 ( 6.2) | 39 ( 7.3) | 94 ( 7.5) | 1.18 | 0.80-1.76 | 1.04 | 0.69-1.57 | 1.38 | 0.91-2.09 | 1.14 | 0.73-1.76 |
| 6-10 | 44 ( 6.3) | 29 ( 5.4) | 86 ( 6.8) | 1.32 | 0.89-1.96 | 1.02 | 0.67-1.56 | 1.12 | 0.71-1.78 | 0.77 | 0.46-1.28 |
| 11-15 | 58 ( 8.3) | 36 ( 6.7) | 90 ( 7.1) | 1.75 | 1.22-2.51 | 1.25 | 0.85-1.84 | 1.45 | 0.95-2.22 | 1.00 | 0.63-1.58 |
| 16-20 | 49 ( 7.0) | 45 ( 8.4) | 65 ( 5.2) | 1.98 | 1.33-2.97 | 1.33 | 0.87-2.05 | 2.42 | 1.59-3.69 | 1.48 | 0.94-2.34 |
| 21-25 | 41 ( 5.9) | 31 ( 5.8) | 48 ( 3.8) | 2.31 | 1.49-3.61 | 1.67 | 1.04-2.68 | 2.46 | 1.51-3.99 | 1.92 | 1.14-3.26 |
| >25 | 208 (29.8) | 159 (29.8) | 170 (13.5) | 3.35 | 2.59-4.33 | 2.04 | 1.54-2.71 | 3.63 | 2.75-4.81 | 2.00 | 1.47-2.73 |
| p-trend |  |  |  | <0.01 |  | <0.01 |  | <0.01 |  | <0.01 |  |
| Cigarette Age at Start^＃^ |  |  |  |  |  |  |  |  |  |  |  |
| Never | 255 (36.4) | 195 (36.5) | 707 (56.1) | 1.00 |  |  |  | 1.00 |  | 1.00 |  |
| >35 | 3 ( 0.4) | 7 ( 1.3) | 6 ( 0.5) | 1.38 | 0.34-5.61 | 0.83 | 0.18-3.76 | 4.62 | 1.50-14.2 | 2.09 | 0.63-6.96 |
| 31-35 | 6 ( 0.9) | 8 ( 1.5) | 9 ( 0.7) | 1.74 | 0.61-4.98 | 1.24 | 0.40-3.83 | 3.93 | 1.49-10.4 | 3.13 | 1.09-9.02 |
| 26-30 | 15 ( 2.2) | 12 ( 2.3) | 34 ( 2.7) | 1.18 | 0.63-2.22 | 1.17 | 0.61-2.26 | 1.39 | 0.70-2.75 | 1.32 | 0.63-2.73 |
| 21-25 | 102 (14.6) | 71 (13.3) | 111 ( 8.8) | 2.44 | 1.79-3.32 | 1.89 | 1.36-2.63 | 2.34 | 1.66-3.30 | 1.79 | 1.23-2.61 |
| 16-20 | 258 (36.9) | 200 (37.5) | 334 (26.5) | 2.05 | 1.64-2.55 | 1.42 | 1.11-1.80 | 2.15 | 1.69-2.73 | 1.37 | 1.05-1.79 |
| <=15 | 61 ( 8.7) | 41 ( 7.7) | 60 ( 4.8) | 2.67 | 1.81-3.95 | 1.31 | 0.86-2.01 | 2.22 | 1.44-3.45 | 1.03 | 0.64-1.66 |
| p-trend |  |  |  | <0.01 |  | <0.01 |  | <0.01 |  | 0.03 |  |
| **Total** | **701** | **534** | **1262** |  |  |  |  |  |  |  |  |

^†^Adjusted for age and study region

^‡^ Adjusted for age, study region, ethnicity, education and family history of NPC

^§^ Data for cigarette smoking duration was missing for 1 prospective NPC case

^¶^ Data for cigarette smoking intensity was missing for 2 prospective NPC cases and 2 controls

^＆^ Data for cigarette pack-yrs was missing for 3 prospective NPC cases and 2 controls

^＃^ Data for cigarette age at start was missing for 1 prospective NPC case and 1 controls

Supplemental Table 3. Characteristics of EBV Seropositive and Seronegative Male Controls - NPC Multicenter Case Control Study in Taiwan

| Risk Factor | # EBV VCA + (%) | # EBV VCA - (%) | X(2) p-Value | # EBV EBNA/EA + (%) | # EBV EBNA/EA - (%) | X(2) p-Value |
| --- | --- | --- | --- | --- | --- | --- |
| Age |  |  | 0.52 |  |  | <0.01 |
| <40 | 33 (11.1) | 265 (88.9) |  | 56 (18.7) | 243 (81.3) |  |
| 40-49 | 28 ( 7.9) | 328 (92.1) |  | 33 ( 9.2) | 324 (90.8) |  |
| 50-59 | 35 ( 9.9) | 318 (90.1) |  | 25 ( 7.1) | 328 (92.9) |  |
| 60-69 | 25 (10.7) | 209 (89.3) |  | 21 ( 8.9) | 214 (91.1) |  |
| Study Region ^†^ |  |  | 0.54 |  |  | 0.63 |
| Northern Taiwan | 106 ( 9.6) | 996 (90.4) |  | 122 (11.0) | 983 (89.0) |  |
| Central Taiwan | 15 (11.3) | 118 (88.7) |  | 13 ( 9.8) | 120 (90.2) |  |
| Unknown | 0 | 6 (100.0) |  | 0 | 6 (100.0) |  |
| Education years |  |  | 0.42 |  |  | 0.78 |
| <=9 | 15 (11.1) | 120 (88.9) |  | 14 (10.2) | 123 (89.8) |  |
| 10-12 | 38 (11.2) | 302 (88.8) |  | 34 (10.0) | 306 (90.0) |  |
| >12 | 68 ( 8.9) | 698 (91.1) |  | 87 (11.3) | 680 (88.7) |  |
| Ethnicity ^‡^ |  |  | 0.37 |  |  | 0.56 |
| Taiwanese | 88 ( 9.9) | 801 (90.1) |  | 91 (10.2) | 801 (89.8) |  |
| Hakka | 13 (11.0) | 105 (89.0) |  | 17 (14.4) | 101 (85.6) |  |
| Mainlander | 18 ( 8.0) | 207 (92.0) |  | 26 (11.6) | 199 (88.4) |  |
| Others | 2 (25.0) | 6 (75.0) |  | 1 (12.5) | 7 (87.5) |  |
| Family History of NPC |  |  | 0.90 |  |  | 0.20 |
| No | 116 ( 9.8) | 1071 (90.2) |  | 132 (11.1) | 1058 (88.9) |  |
| Yes | 5 ( 9.3) | 49 (90.7) |  | 3 ( 5.6) | 51 (94.4) |  |
| Salted Fish @ Young Age^§^ |  |  | 0.73 |  |  | 0.28 |
| No | 106 ( 9.6) | 1004 (90.4) |  | 122 (11.0) | 990 (89.0) |  |
| Yes | 5 ( 8.2) | 56 (91.8) |  | 4 ( 6.6) | 57 (93.4) |  |
| **Total** | **121 ( 9.8)** | **1120 (90.2)** |  | **135 (10.9)** | **1109 (89.1)** |  |

^†^ Data for study region was missing for 6 subjects

^‡^ Data for ethnicity was missing for 1 subject

^§^ Data for salted fish at young age was missing for 70 subjects with anti-EBV VCA IgA and 71 subjects with anti-EBV EBNA1-EA IgA

Supplements Table 4. Distribution of EBV Levels Among EBV Positive Controls (Males Only)

| Smoking | VCA | | | | | | EBNA1/EA | | | | | |
| --- | --- | --- | --- | --- | --- | --- | --- | --- | --- | --- | --- | --- |
|  | N (%) Positive | Among Positives: | | | | | N (%) Positive | Among Positives: | | | | |
|  |  | Median | SD | Range | IQR | p-value* |  | Median | SD | Range | IQR | p-value* |
| Never | 58 (8.4) | 1.57 | 0.70 | 3.15 | 0.86 | 0.87 | 80 (11.5) | 4.14 | 1.69 | 12.1 | 1.89 | 0.69 |
| Former | 21 (8.4) | 1.39 | 0.77 | 2.44 | 0.50 |  | 18 ( 7.2) | 3.95 | 2.17 | 7.89 | 0.99 |  |
|  |  |  |  |  |  |  |  |  |  |  |  |  |
| Quit >10 years ago | 10 ( 7.5) | 1.40 | 0.84 | 2.44 | 0.32 | 0.91 | 12 ( 9.0) | 4.03 | 1.74 | 6.34 | 1.09 | 0.73 |
| Quit 6-10 years ago | 5 ( 8.3) | 1.18 | 0.97 | 2.27 | 0.47 |  | 5 ( 8.3) | 3.80 | 3.36 | 7.89 | 0.96 |  |
| Quit <=5 years ago | 6 (10.9) | 1.47 | 0.59 | 1.68 | 0.48 |  | 1 (1.8) | 3.83 | - | - | - |  |
|  |  |  |  |  |  |  |  |  |  |  |  |  |
| Current | 42 (14.0) | 1.37 | 1.27 | 6.68 | 1.12 |  | 37 (12.3) | 4.20 | 1.50 | 6.10 | 1.84 |  |
|  |  |  |  |  |  |  |  |  |  |  |  |  |
| <=10 years | 4 (12.9) | 1.42 | 1.47 | 3.19 | 1.61 | 0.92 | 4 (12.9) | 3.81 | 0.47 | 1.14 | 0.66 | 0.75 |
| 11-20 years | 10 (12.7) | 1.30 | 2.01 | 6.67 | 0.75 |  | 15 (19.0) | 4.24 | 1.61 | 4.58 | 2.91 |  |
| >20 years | 28 (14.8) | 1.41 | 0.91 | 3.79 | 1.15 |  | 18 ( 9.4) | 4.09 | 1.55 | 6.10 | 1.15 |  |
|  |  |  |  |  |  |  |  |  |  |  |  |  |
| <= 15 cigarettes | 17 (10.9) | 1.41 | 1.16 | 3.80 | 1.03 | 0.27 | 18 (11.5) | 4.27 | 0.89 | 3.06 | 1.26 | 0.09 |
| 16-25 cigarettes | 15 (15.3) | 1.51 | 1.67 | 6.60 | 1.06 |  | 8 ( 8.1) | 6.63 | 2.25 | 6.10 | 3.82 |  |
| >25 cigarettes | 10 (22.2) | 1.13 | 0.59 | 1.53 | 0.90 |  | 11 (24.4) | 3.51 | 1.06 | 3.59 | 1.04 |  |

* Use Kruskal-Wallis test
